# Supplementary figures and images for: Do children allocated to different methods of complementary feeding introduction have distinct food preferences and flavor acceptance in the first years of life? A randomized clinical trial
Source: PLoS One. 2025 Nov 14;20(11):e0335592. doi: 10.1371/journal.pone.0335592 (PMC12617864; doi:10.1371/journal.pone.0335592)

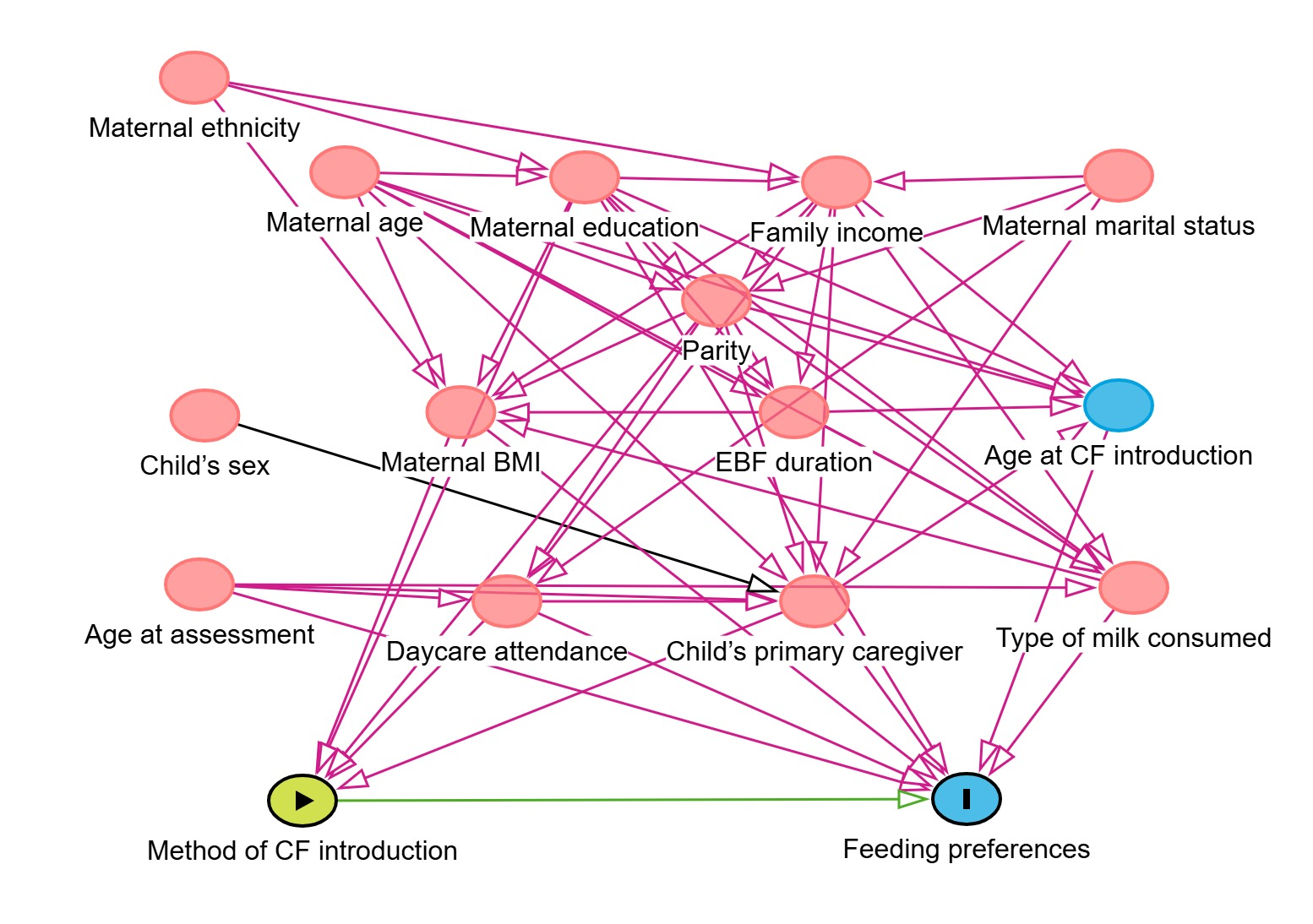

Supplement: S1 Fig — Legend: CF = complementary feeding; BMI = Body Mass Index; EBF = exclusive breastfeeding. (TIFF) [file pone.0335592.s001.tiff]

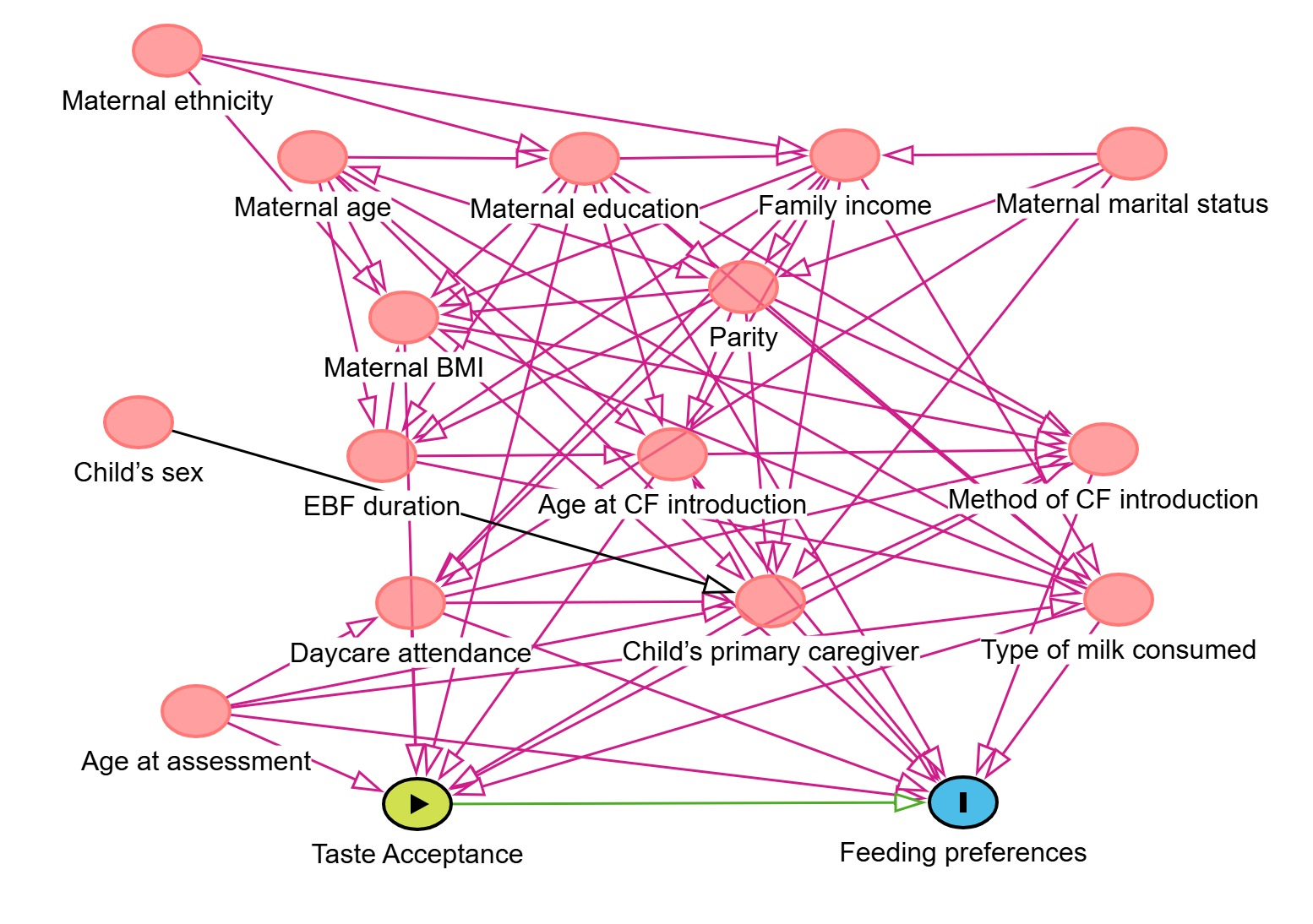

Supplement: S2 Fig — Legend: CF = complementary feeding; BMI = Body Mass Index; EBF = exclusive breastfeeding. (TIFF) [file pone.0335592.s002.tiff]
