# Supplementary material for: Do children allocated to different methods of complementary feeding introduction have distinct food preferences and flavor acceptance in the first years of life? A randomized clinical trial
Source: PLoS One. 2025 Nov 14;20(11):e0335592. doi: 10.1371/journal.pone.0335592 (PMC12617864; doi:10.1371/journal.pone.0335592)
Supplement: S1 Table — (DOCX) [file pone.0335592.s003.docx]

| **Supplementary Table 1. Comparison of the characteristics baseline of completers and non-completers of the food preference questionnaire and the taste acceptance test in the study.** | | | | | | | |
| --- | --- | --- | --- | --- | --- | --- | --- |
| **Variables** | **Total**  **(n=140)** | **Feeding Preferences Questionnaire** | | | **Taste Acceptance Test** | | |
|  |  | **Completers**  **(n=132)** | **Non-completers**  **(n=8)** | ***p*** | **Completers**  **(n=92)** | **Non-completers**  **(n=48)** | ***p*** |
| **CF method**  PLW  BLISS  Mixed | 45 (32.1)  48 (34.3)  47 (33.6) | 44 (97.8)  46 (95.8)  42 (89.4) | 1 (2.2)  2 (4.2)  5 (10.6) | 0.222 | 31 (68.9)  34 (70.8)  27 (57.4) | 14 (31.1)  14 (29.2)  20 (42.6) | 0.345 |
| **Maternal ethnicity**  White  Non-white | 120 (85.7)  20 (14.3) | 112 (93.3)  20 (100.0) | 8 (6.7)  0 (0.0) | 0.602 | 78 (65.0)  14 (70.0) | 42 (35.0)  6 (30.0) | 0.663 |
| **Marital status**  With partner  Without partner | 118 (84.3)  22 (15.7) | 113 (95.8)  19 (86.4) | 5 (4.2)  3 (13.6) | 0.112 | 80 (67.8)  12 (54.6) | 38 (32.2)  10 (45.4) | 0.229 |
| **Maternal education (years)** | 18.0 [15.0; 20.0] | 18.0 [15.0; 20.0] | 17.5 [14.5; 18.5] | 0.675 | 17.5 [14.0; 20.0] | 18.0 [15.5; 21.0] | 0.167 |
| **Family income (BRL)*** | 6,000.0  [4,000.0; 10,000.0] | 6,000.0  [4,000.0; 10,000.0] | 4,500.00  [3,250.0; 9,000.0] | 0.446 | 6,000.0  [4,000.0; 10,000.0] | 7,000.0  [4,000.0; 10,500.0] | 0.446 |
| **Parity**  Primiparous  Multiparous | 113 (80.7)  27 (19.3) | 108 (95.6)  24 (88.9) | 5 (4.4)  3 (11.1) | 0.182 | 71 (62.8)  21 (77.8) | 42 (37.2)  6 (22.2) | 0.142 |
| **Sex of the child**  Male  Female | 68 (48.6)  72 (51.4) | 65 (95.6)  67 (93.1) | 3 (4.4)  5 (6.9) | 0.719 | 45 (66.2)  47 (65.3) | 23 (33.8)  25 (34.7) | 0.911 |
| **Maternal age (years)** | 34.0 [29.5; 36.0] | 33.5 [30.0; 36.0] | 35 [25.5; 36.5] | 0.964 | 34.0 [31.0; 36.0] | 32.5 [26.0; 36.0] | 0.362 |
| **Maternal BMI***  Eutrophy  Overweight/Obesity | 51 (47.2)  57 (52.8) | 49 (96.1)  53 (93.0) | 2 (3.9)  4 (7.0) | 0.682 | 38 (74.5)  36 (63.2) | 13 (25.5)  21 (36.8) | 0.205 |
| **EBF time (days)** | 180.0 [150.0; 180.0] | 180.0 [150.0; 180.0] | 171.0 [90.0; 180.0] | 0.277 | 180.0 [151.5; 180.0] | 180.0 [120.0; 180.0] | 0.508 |
| **CF introduction (days)** | 180.0 [180.0; 180.0] | 180.0 [180.0; 180.0] | 180.0 [180.0; 184.0] | 0.698 | 180.0 [180.0; 180.0] | 180.0 [180.0; 180.0] | 0.689 |
| Legend: n = number of participants; PLW = Parent-Led Weaning; BLISS = Baby-Led Introduction to SolidS; BMI = body-mass-index; EBF = exclusive breastfeeding; CF = complementary feeding; BF = breastfeeding.  Statistical tests: Pearson's Chi-square or Fisher's exact, for qualitative variables (expressed with n(%)), and Mann Whitney test for quantitative variables (expressed with median [P25; P75]).  The *n* of some variables, highlighted with an asterisk (*), may not reach the total due to missing data. | | | | | | | |
