# Supplementary material for: Do children allocated to different methods of complementary feeding introduction have distinct food preferences and flavor acceptance in the first years of life? A randomized clinical trial
Source: PLoS One. 2025 Nov 14;20(11):e0335592. doi: 10.1371/journal.pone.0335592 (PMC12617864; doi:10.1371/journal.pone.0335592)
Supplement: Attachment — Submitted filename: Response to Reviewers Plos One_09.01.docx [file pone.0335592.s007.docx]

Porto Alegre (RS), Brazil, September 01, 2025

Dear Academic Editor and Reviewers,

The authors would like to thank you for the careful evaluation of our manuscript entitled “**Do children allocated to different methods of complementary feeding introduction have distinct food preferences and flavor acceptance in the first years of life? A randomized clinical trial**” and for the comments and suggestions provided. We appreciate the opportunity to revise our manuscript and have addressed all the points raised in the review process.

Below, we provide a point-by-point response to the journal requirements and each comment from the reviewers and the academic editor. Our responses are presented in blue and indicate where the changes have been made in the revised manuscript (file: “Revised Manuscript with Track Changes”).

We believe that the changes made have significantly improved the manuscript and hope that our revised version meets the journal’s publication criteria and is now suitable for publication in PLOS ONE.

Finally, we would like to note that one additional author has been included in this version of the manuscript, given her substantial contribution to the construction of the statistical models required for the revision, as well as her participation in writing, editing and reviewing the manuscript. We would also like to respectfully inform that, for reasons of logistical convenience, the corresponding author is the undersigned of this letter.

Kind regards,

Cátia Regina Ficagna

On behalf of all co-authors

**JOURNAL REQUIREMENTS:**

When submitting your revision, we need you to address these additional requirements.

**1.** Please ensure that your manuscript meets PLOS ONE's style requirements, including those for file naming. The PLOS ONE style templates can be found at <https://journals.plos.org/plosone/s/file?id=wjVg/PLOSOne_formatting_sample_main_body.pdf> and <https://journals.plos.org/plosone/s/file?id=ba62/PLOSOne_formatting_sample_title_authors_affiliations.pdf>

**Response:** We review the manuscript formatting according to the provided style templates, including file naming, to ensure it fully meets PLOS ONE style requirements.

**2.** Thank you for submitting your clinical trial to PLOS ONE and for providing the name of the registry and the registration number. The information in the registry entry suggests that your trial was registered after patient recruitment began. PLOS ONE strongly encourages authors to register all trials before recruiting the first participant in a study.

As per the journal’s editorial policy, please include in the Methods section of your paper:
1) your reasons for your delay in registering this study (after enrolment of participants started);
2) confirmation that all related trials are registered by stating: “The authors confirm that all ongoing and related trials for this drug/intervention are registered”.

**Response:** After study approval by the Research Ethics Committee, the RCT was registered at https://ensaiosclinicos.gov.br/rg/RBR-229scm, identifier [RBR-229scm U1111-1226-9516]. Due to administrative and logistical challenges during the initial phase of the study’s preparation, there was a delay in registering this clinical trial.. Specifically, the registration process took longer than anticipated due to the time required to obtain internal approvals and complete the study documentation. This explanation has now been included under “Registration” in the Methods section. In addition, we confirm that all related trials have been registered, including the requested declaration, as follows:

**“Registration**

*After study approval by the Research Ethics Committee, the RCT was registered at https://ensaiosclinicos.gov.br/rg/RBR-229scm, identifier [RBR-229scm U1111-1226-9516]. The authors confirm that all ongoing and related trials for this intervention are registered.”*

**3.** We note that the grant information you provided in the ‘Funding Information’ and ‘Financial Disclosure’ sections do not match. When you resubmit, please ensure that you provide the correct grant numbers for the awards you received for your study in the ‘Funding Information’ section.

**Response:** Thank you for pointing this out. Indeed, the grant number from CNPq (grant number: 20190230) was missing in the Financial Disclosure section. We have now added the correct grant number during the resubmission and ensured that the information matches in both the “Funding Information” and “Financial Disclosure” sections.

**4.** Thank you for stating the following financial disclosure:

“This research was supported by the Conselho Nacional de Desenvolvimento Científico e Tecnológico (CNPq), Brazil; Fundo de Incentivo à Pesquisa e Eventos (FIPE), Hospital de Clínicas de Porto Alegre, Brazil [grant number 2019-0540]; Coordenação de Aperfeiçoamento de Pessoal de Nível Superior (CAPES), Brazil [grant number 407426/2021-3].”

Please state what role the funders took in the study.  If the funders had no role, please state: "The funders had no role in study design, data collection and analysis, decision to publish, or preparation of the manuscript." If this statement is not correct you must amend it as needed.

Please include this amended Role of Funder statement in your cover letter; we will change the online submission form on your behalf.

**Response:** We thank you for your comment. We confirmed that the funders had no role in study design, data collection and analysis, decision to publish, or preparation of the manuscript, and this will be confirmed at the time of submission.

**5.** In the online submission form, you indicated that “Data will be made available on request.”. 
All PLOS journals now require all data underlying the findings described in their manuscript to be freely available to other researchers, either:

1. In a public repository,

2. Within the manuscript itself, or

3. Uploaded as supplementary information.

This policy applies to all data except where public deposition would breach compliance with the protocol approved by your research ethics board. If your data cannot be made publicly available for ethical or legal reasons (e.g., public availability would compromise patient privacy), please explain your reasons on resubmission and your exemption request will be escalated for approval.

**Response:** We thank you for your comment. The dataset generated and analyzed during the current study will be made publicly available as supplementary information upon resubmission, in accordance with PLOS ONE data availability requirements. No ethical or legal restrictions prevent the sharing of this dataset.

**6.** Please include captions for your Supporting Information files at the end of your manuscript, and update any in-text citations to match accordingly. Please see our Supporting Information guidelines for more information: <http://journals.plos.org/plosone/s/supporting-information>.

**Response:** We thank you for your comment. We would include full captions for all Supporting Information files at the end of the manuscript and update the in-text citations accordingly, in accordance with the PLOS ONE Supporting Information guidelines.

**7.** We note that the original protocol that you have uploaded as a Supporting Information file contains an institutional logo. As this logo is likely copyrighted, we ask that you please remove it from this file and upload an updated version upon resubmission.

**Response:** We thank you for your comment. We would remove the institutional logo from the original protocol file and upload an updated version as Supporting Information upon resubmission.

**REVIEWERS' COMMENTS**

**REVIEWER #1:**

**General Comments:** This manuscript addresses a relevant and timely research question, examining how different methods of complementary food introduction—Parent-Led Weaning (PLW), Baby-Led Introduction to Solids (BLISS), and a Mixed approach—affect food preferences and taste acceptance among young children. Given the increasing interest from both clinicians and parents regarding early dietary influences on later eating behaviors, the study's objective is particularly valuable. The authors have employed a robust randomized clinical trial (RCT) design and clearly defined interventions, which significantly strengthen the methodological rigor. The manuscript is generally well-structured, clearly written, and includes appropriate references to prior literature. Methodological details, including randomization, blinding, and statistical analyses (robust Poisson regression adjusted via Directed Acyclic Graph \[DAG] and intention-to-treat analyses), are suitably described. The combination of parent-reported feeding preferences and an objective laboratory taste acceptance test is commendable, adding depth and rigor to the findings.

However, several issues warrant attention to enhance clarity, accuracy, and appropriate interpretation. Below, I outline key points that require revisions, categorized as major and minor issues.

**Major Issues:**

**1)** Clarification of Primary and Secondary Outcomes: It is currently unclear which specific outcome measures were identified as primary or secondary prior to study initiation. The manuscript evaluates multiple outcomes across taste categories and groups, raising the potential for Type I error due to multiple comparisons. It would improve clarity to explicitly state the primary outcome(s) designated a priori or clearly acknowledge that the analyses are exploratory. Additionally, authors should discuss potential risks of Type I errors explicitly, especially given that significant findings were limited to sour taste preferences.

**Response:** We appreciate the comment. As described in the randomized clinical trial protocol (Nunes et al., 2021), the primary outcomes were z-scores of anthropometric measurements (weight for age, weight for length, length for age, and BMI for age z-scores) to investigate child growth and nutritional status. Secondary outcomes included food and flavor preferences, among other outcomes (such as choking prevalence, dietary variety, child and parent feeding behavior, iron deficiency, oral hygiene behavior, dental caries, dental development, gingival health status, the prevalence of functional constipation, maternal perception of the methods of CF introduction, and prevalence of child eating disorders). To clarify this, we revised the Methods section of the manuscript and included an “Outcomes” subsection to explicitly indicate that food and flavor preferences were assessed as a priori-defined secondary outcomes, as follows:

*“****Outcomes***

*The RCT had as primary outcomes the z-scores of anthropometric measurements (weight for age, weight for length, length for age, and BMI for age z-scores) [9,10] to investigate child growth and nutritional status [18]. The secondary outcomes, defined a priori in the RCT, included, among others (prevalence of choking, dietary variety, child and parental eating behavior, iron deficiency, oral hygiene behavior, dental caries, dental development, gingival health, prevalence of functional constipation, maternal perception of the methods of CF introduction, and prevalence of childhood eating disorders), food and flavor preferences [9,10], which were investigated as outcomes in the present study.”*

*18. Ficagna CR, Magalhães EIDS, Chacón AB, Moreira PR, Neves RO, Nunes LM, Bernardi JR. Impact of complementary feeding methods on infant nutritional status: A randomized clinical trial. Nutrition. 2025 Jul 11;140:112908. doi: 10.1016/j.nut.2025.112908. Epub ahead of print. PMID: 40782569.*

Regarding the potential risks of Type I errors, we included in the Discussion section a note about the risk of these errors due to multiple comparisons, highlighting that the significant findings were limited to the sour taste, as below:

*“On the other hand, given the multiple comparisons performed, there is an increased risk of Type I error. This is particularly relevant as significant findings were limited to sour taste preferences, which should be interpreted with caution.”*

**2)** Details and Transparency of Statistical Analysis: The authors employed robust Poisson regression appropriately, but clarity regarding adjusted versus unadjusted results is needed. The manuscript should explicitly state the covariates used in adjusted models and clearly indicate in the tables and results section which comparisons were adjusted or unadjusted. This transparency will enhance rigor and interpretability.
**Response:** We appreciate the comment. As requested, we revised the text of the Statistical Analyses in the Methods section to clarify the covariates used in the adjusted models. We also revised the text of the Results section, as well as Tables 2 and 4, to clearly indicate the adjusted and unadjusted results. Below is the revised text of the Methods and Results sections:

**Methods section:**

***“Statistical Analysis***

*All analyses were performed by intention-to-treat. The database was created utilizing the SPSS® Program – Statistical Package for the Social Sciences version 29.0, with double entry and subsequent validation.*

*Categorical variables were described using absolute and relative frequencies [(n (%)] and numeric variables were described as measures of central tendency and dispersion (mean and standard deviation; median and P25 - P75). The distribution of numeric variables was assessed through visual inspection of histograms, verification of asymmetry and kurtosis measures, as well as the application of the Shapiro-Wilk test. To compare the sociodemographic, nutritional, and childcare-related characteristics of the study sample between the intervention groups (PLW, BLISS and Mixed), Pearson's chi-square test was used for the proportions of categorical variables, and the Mann-Whitney test was used for the medians of numerical variables. The Mann-Whitney test was also used to compare the medians of the volume consumed in the taste acceptance test according to the infant's reaction (Acceptance and rejection) to the same substances. A significance level of 5% (p ≤ 0.05) was considered for all analyses.*

*Poisson regression analysis with robust variance was used to estimate prevalence ratios and their 95% confidence intervals for the associations between the method of CF introduction and food preferences according to the predominant taste, as well as between food preferences according to the predominant taste and reactions in the taste acceptance test. To control for confounding, Poisson regression analysis was adjusted for potential confounding factors. A minimum adjustment set of variables to control for confounding in each association evaluated was identified based on a directed acyclic graph (DAG) constructed using the online DAGitty 3.2 software, applying the backdoor criterion [24]. The DAG for estimating the total effect of the method of CF introduction on food preferences according to the predominant taste indicated the need for adjustment for: Maternal education, parity, maternal BMI, daycare attendance and child’s primary caregiver (Supplementary Figure 1) . The DAG for estimating the total effect of the food preferences according to the predominant taste on reactions in the taste acceptance test, in turn, indicated the need for adjustment for: Maternal education, maternal BMI, age at CF introduction, method of CF introduction, age at the application of the food preferences questionnaire and taste acceptance, daycare attendance, child’s primary caregiver and type of milk consumed (Supplementary Figure 2).”*

**Results section:**

**Lines 305 to 314:**

*“The crude and adjusted prevalence ratios of the association between the method of CF introduction and the food preferences according to the predominant taste are shown in Table 2. In adjusted analysis, the prevalence of preferences for foods with a predominant sour taste were 23% higher (Crude PR 1.23, 95% CI 1.03 - 1.47, p=0.020) in the Mixed method compared to the PLW, however, after adjusting for covariates, this association did not remain statistically significant (Adjusted PR 1.15, 95% CI 0.94 - 1.41, p=0.173). The preference for foods with other predominant tastes didn’t show associations with the method of CF introduction, neither in the unadjusted nor in the adjusted analysis.”*

**Lines 331 to 337:**

*“The crude and adjusted PR of the association between the food preferences according to the predominant taste and the reactions in the taste acceptance test are shown in Table 4. In unadjusted analysis, food preferences related to the bitter taste are associated with higher acceptance of the solution with the same taste (Crude PR 1.12, 95% CI 1.01 - 1.25, p=0.046). However, in adjusted analysis, this association loses its significance (Adjusted PR 1.16, 95% CI 0.99 – 1.37, p=0.069).”*

**3)** Handling of Missing Data and Attrition: Attrition, particularly in the Taste Acceptance Test (with approximately 34% non-completion), raises concerns about potential bias. The authors should detail reasons for attrition clearly within the participant flow diagram and address explicitly whether attrition was balanced across groups. Additionally, discuss whether attrition may have introduced bias, potentially influencing outcomes. A brief comparison of baseline characteristics between completers and non-completers would further strengthen methodological transparency.

**Response:** We appreciate the feedback. As requested, we have included the reasons for attrition within the participant flow diagram. We also performed an analysis comparing the baseline characteristics between completers and non-completers of the food preferences questionnaire and the taste acceptance test. The results are presented in Supplementary Table 1 and show that there were no statistically significant differences between the groups. Finally, we added a sentence in the discussion section to inform that, based on the analyses performed, the data loss was not differential and therefore could not have introduced bias, potentially influencing the results, as below:

*“There were participant losses in the food preferences questionnaire and taste acceptance test. However, we performed an analysis comparing the baseline characteristics between completers and non-completers of the food preferences questionnaire and the taste acceptance test and no statistically significant differences were observed between the groups (Supplementary Table 1). Therefore, attrition bias is unlikely to influence the results.”*

**4)** Measurement Validity and Limitations: Additional detail on the structure, validity, and potential biases of the Feeding Preferences Questionnaire is necessary. The subjective nature of parent-reported preferences and the possibility of reporting bias should be explicitly addressed. Furthermore, the authors should clarify the limitations of the Taste Acceptance Test, emphasizing that laboratory responses may not fully capture everyday food acceptance behaviors.
**Response:** We thank you for your comment. We have revised the Methods section to make the description clearer and added, in the limitations of the Discussion section, the possible biases that the Food Preferences Questionnaire and the Taste Acceptance Test may present, as below:

**Methods section:**

*“The Food Preferences Questionnaire was translated from Schwartz et al. [19], a previously validated instrument for assessing children’s food acceptance, with examples of food groups according to their predominant taste (sweet, sour, salty, umami, and bitter); and was analyzed according to a 5-point scale varying between strong rejection and strong acceptance. An example of a food that was not commonly consumed in Brazil was removed from the test at the time of translation.”*

**Discussion section:**

*“The Food Preferences Questionnaire, as a self-reported questionnaire, may be subject to the caregiver’s subjective interpretation of the child’s acceptance or rejection of foods. For the Taste Acceptance Test, factors such as the testing environment, use of unfamiliar utensils, offer of artificial solutions instead of foods, and absence of usual social mealtime influences may affect reactions. Both instruments capture specific aspects of food acceptance and preference, but do not replace observation in natural settings.”*

**5)** Interpretation of Findings and Practical Significance: The authors' interpretation of the findings should be tempered. Given the limited number of significant differences (only sour taste preference showed significance), conclusions suggesting broad impacts of feeding methods should be revised to reflect the modest and limited scope of the observed effects. Additionally, the manuscript should discuss the potential practical implications of these small observed differences clearly and cautiously.

Response: We thank you for your comment and agree that the results should be interpreted with caution. We have modified the first paragraph of the Discussion, as well as the Conclusion, as below:

***“Discussion***

*The PLW method of CF introduction presented a lower preference for sour-tasting foods compared with the Mixed method in the crude analysis. However, this association did not remain statistically significant after the adjusted analysis. No associations were observed for foods with other predominant tastes, either in the crude or adjusted analyses. The findings should therefore be interpreted with caution, as they suggest only a modest influence of CF methods on taste preferences. Moreover, an association between the Taste Acceptance Test and the Food Preferences Questionnaire was observed in the crude analysis for the bitter taste; nevertheless, these results may serve as an indication warranting further research with a larger sample on how complementary feeding methods could influence children’s taste preferences.”*

*“In the complementary analysis of this study, a crude association was observed between bitter taste preferences and higher acceptance of the same flavor in the Taste Acceptance Test. However, this association did not remain significant after adjustment.”*

***“Conclusion***

*Infants allocated to the Mixed group showed a greater preference for the sour taste compared with the PLW group. However, this association did not remain statistically significant after the adjusted analysis and should therefore be interpreted with caution. The association observed between bitter taste preferences and the acceptance of solutions with the same taste also did not remain after adjustment, reinforcing the preliminary nature of these findings. Future longitudinal research is needed to better explore infant feeding behaviors in relation to adherence to CF methods and to include foods specific to the Brazilian cultural context.*

**6)** Sample Size and Power Considerations: Clarify explicitly the primary endpoint used for the original power calculation. If taste preference was not the primary outcome, clearly acknowledge the limitations this places on detecting subtle between-group differences in taste acceptance. This clarification will aid in interpreting the largely null findings for most taste categories.

**Response:** We thank the reviewer for this comment. We have revised the Sample Size section, as below:

*“The sample size calculation was performed for the primary outcome of the randomized clinical trial (anthropometric z-scores), as described in a previous publication and based on prior studies on the subject [20, 21]. Considering a unit standard deviation equal to 1, with 80% power and a 5% significance level, the calculation resulted in 48 mother–infant pairs for each of the three intervention groups, totaling 144 pairs. The WinPepi® software was used for the calculations.”*

In addition, as described in the RCT protocol (Nunes et al., 2021), the primary outcomes used for the sample size and power calculation were anthropometric z-scores (weight-for-age, weight-for-length, length-for-age, and BMI-for-age), aimed at assessing child growth and nutritional status. The secondary outcomes, defined a priori, included food and flavor preferences among several other variables. To clarify this point, we revised the Methods section and added a subsection, ‘Outcomes,’ explicitly indicating that taste preferences were assessed as secondary outcomes.

*“****Outcomes***

*The RCT had as primary outcomes the z-scores of anthropometric measurements (weight for age, weight for length, length for age, and BMI for age z-scores) [9,10], which have already been investigated in another publication [18]. The secondary outcomes, defined a priori in the RCT, included, among others (prevalence of choking, dietary variety, child and parental eating behavior, iron deficiency, oral hygiene behavior, dental caries, dental development, gingival health, prevalence of functional constipation, maternal perception of methods of CF introduction, and prevalence of childhood eating disorders), food and flavor preferences [9,10], which were investigated as outcomes in the present study.”*

**7)** Adherence to Assigned Feeding Methods: The manuscript should briefly summarize adherence to feeding methods as previously published by the authors, clearly discussing how actual adherence levels may have influenced study outcomes. Recognizing variability in adherence provides essential context for interpreting the subtle differences observed.

**Response:** We thank you for your comment. We have added a paragraph in the Discussion section addressing adherence to the methods of CF introduction, as published by Sanini Belin et al., 2023, as below:

*“As previously published in a specific study on adherence to complementary feeding methods from this RCT [15], overall adherence to the PLW, BLISS, and mixed methods was 34.1% at 7 months, 28.5% at 9 months, and 34.1% at 12 months. The mixed method showed the highest adherence at all ages (69.0%, 55.8%, and 78.6%, respectively; p<0.001). In addition, a large proportion of families initially allocated to PLW and BLISS migrated to the mixed method during the first year of life (60% and 72.9% regarding feeding mode and 97.8% and 100% regarding food consistency, respectively). This variability in adherence may have reduced the differences between groups, which helps explain the results observed in taste preferences.”*

**Reference:**

Sanini Belin CH, Nunes LM, Ficagna CR, Neves RO, Moreira PR, Bernardi JR. Adherence to different complementary feeding methods in the first year of life: A randomized clinical trial. Cook EJ, editor. PLoS ONE. 2023;18:e0289696. doi:10.1371/journal.pone.0289696.

**Minor Issues:**

**1)** Language and Terminology:

**1.1)** Replace "mensal" with "monthly" for clarity and correct terminology.

**Response:** Thank you for your comment. We have replaced “mensal” with “monthly” for clarity and correct terminology, as below:

*“The descriptive data of the sample are presented in Table 1. Of the 132 mothers, 112 (84.8%) considered themselves of white ethnicity, 113 (85.6%) lived with their partner at the moment of the study, had a median of 18 [15; 20] years of study, and had a median monthly family income of R$6,000.00 [4,000.00; 10,000.00]. Moreover, 108 (81.8%) were primiparous, and half (50.8%) of the infants were female.”*

**1.2)** Clarify clearly on first mention that the "Mixed" method involved initially using BLISS but switching to spoon-feeding if necessary.

**Response:** We thank you for your comment. We have provided a detailed description of the "Mixed" method in the Intervention section, as below:

*Mixed: This group was instructed to combine the two methods explored above, according to the wishes of the child. The family should initially offer foods using the BLISS technique. If the child showed dissatisfaction or disinterest in food, according to the BLISS technique, they were instructed to offer the food using the PLW technique at the same meal.*

**2)** Data Presentation and Table Corrections: Correct the error in Table 2 (Mixed method salty taste PR value of "0.10" should likely be "1.00"). Verify consistency in decimal places and formatting.
**Response:** Thank you for your observation. In fact, there was a typing error in the value of the RP mentioned, and the correct value is 0.99. Therefore, we corrected the value of this RP in Table 2. Regarding the decimal places, we have standardized them to two digits after the decimal point for the 95% CI values in both Table 2 and Table 4 to ensure consistency in data presentation and formatting.

**3)** Figure and Flowchart: Provide a clear, detailed participant flowchart illustrating numbers randomized, completed, and lost for each group.

**Response:** We thank the reviewer for this suggestion. A detailed participant flowchart has been added to the revised manuscript, illustrating the number of participants randomized, completed, and lost for each group, as below:

**4)** Discussion Contextualization: Emphasize explicitly the transient and limited influence early feeding methods may have on taste preferences by referencing relevant longitudinal studies and aligning findings with existing literature.

**Response:** We thank you for your comment. We have added the following paragraph to the discussion:

*“The influence of methods of CF introduction on taste preferences appears to be transient and limited. Longitudinal studies show that these preferences change during the first years of life [33, 38] and that later factors, such as overall diet quality, may play a greater role than the initial feeding method [39]. Thus, our findings reinforce the idea that the observed effects are modest and temporary.”*

**5)** References and Formatting: Use consistent terminology for "Parent-Led Weaning" and "traditional method" to avoid confusion.

**Response:** We appreciate your comment. For greater clarity, we have revised the terminology throughout the manuscript, maintaining the terms “*Parent-Led Weaning (PLW)”.*

**6)** The manuscript presents valuable and rigorously conducted research with the potential for publication. Addressing the outlined major and minor issues will significantly improve clarity, methodological transparency, and appropriate interpretation, thus aligning the manuscript fully with high-quality standards required for acceptance.

**Response:** We appreciate the recognition of the value and rigor of our research. We have carefully addressed all the major and minor issues outlined to improve the clarity, methodological transparency, and interpretation of our manuscript. We believe these revisions have strengthened the manuscript and brought it into full alignment with the high-quality standards required for publication.

**REVIEWER #2:**

**1)** Abstract: need to spell out the abbreviation of “PR” for the first time.

**Response:** Thank you for your comment. We have spelled out the abbreviation “PR” the first time it appears in the abstract, as below.

*“In unadjusted analysis, prevalence of infants’ preferences for foods with a predominant sour taste was higher in the Mixed method compared to the PLW [Crude prevalence ratio (PR) 1.23, 95% CI 1.03 - 1.47, p=0.020].”*

**2)** Sample size: what test was used? Which one is the primary outcome? The calculation is based on a continuous measure (standard deviation), however, all the outcomes in Table 2 are binary. It is not clear how the 1.4 SD was selected.

**Response:** We thank the reviewer for this comment. We have revised the Sample Size section, as below:

“*The sample size calculation was performed for the primary outcome of the randomized clinical trial (anthropometric z-scores), as described in a previous publication and based on prior studies on the subject [20, 21]. Considering a unit standard deviation equal to 1, with 80% power and a 5% significance level, the calculation resulted in 48 mother–infant pairs for each of the three intervention groups, totaling 144 pairs. The WinPepi® software was used for the calculations.”*

**3)** The p-values need to be adjusted for multiple tests, especially for Table 2.

**Response:** We thank you for the comment. For Table 2, as requested, we included the prevalence ratios and 95% CIs with their respective p-values from the analysis adjusted for covariates. Regarding Table 3, the analyses presented are purely descriptive and intended to summarize the data rather than to formally test hypotheses. Therefore, we did not apply adjustments for multiple comparisons, as the p-values are reported only to aid in data interpretation and not as definitive evidence of statistical associations.
