# Supplementary material for: Do children allocated to different methods of complementary feeding introduction have distinct food preferences and flavor acceptance in the first years of life? A randomized clinical trial
Source: PLoS One. 2025 Nov 14;20(11):e0335592. doi: 10.1371/journal.pone.0335592 (PMC12617864; doi:10.1371/journal.pone.0335592)
Supplement: Attachment — Submitted filename: Response to Reviewers_R2.docx [file pone.0335592.s008.docx]

Porto Alegre (RS), Brazil, September 24, 2025

Dear Academic Editor and Reviewers,

The authors would like to thank you for the careful evaluation of our manuscript entitled “**Do children allocated to different methods of complementary feeding introduction have distinct food preferences and flavor acceptance in the first years of life? A randomized clinical trial**” and for the comments and suggestions provided. We are encouraged that the reviewers considered our previous responses satisfactory.

In this revised version, we have implemented the Minor Editorial Adjustments requested. Below, we provide a point-by-point response to the journal requirements and each comment from the academic editor. Our responses are presented in blue and indicate where the changes have been made in the revised manuscript (file: “Revised Manuscript with Track Changes”).

The authors believe that the changes made have significantly improved the manuscript and hope that our revised version fully meets PLOS ONE’s publication criteria and is now suitable for publication.

Kind regards,

Cátia Regina Ficagna

On behalf of all co-authors

**Minor Editorial Adjustments**

**1.** Ensure consistent tense usage throughout the manuscript, particularly when describing methods (past tense) and results (past tense).

**Response:** As requested, we reviewed the entire manuscript and adjusted the text to ensure consistency of verb tense throughout, using the past perfect tense, particularly in the Methods and Results sections.

**2.** Double-check supplementary figure captions to ensure they are placed correctly and consistently referenced in the main text.

**Response:** We checked the supplementary figure captions and inserted them in the text of the manuscript, following the paragraph in which the figure is first cited.

**3.** Standardize abbreviation usage (e.g., consistently use “CF” for complementary feeding after the first definition).

**Response:** We revised the manuscript text and standardized the abbreviation usage throughout, consistently using “CF” for complementary feeding after its first definition.

**4.** Review for minor typographical errors (spacing, punctuation, en-dash vs. hyphen in numerical ranges).

**Response:** We have reviewed the manuscript for minor typographical errors, including spacing, punctuation, and the correct use of en-dash versus hyphen in numerical ranges, and made necessary corrections.

**5.** Verify reference formatting consistency (especially newer references added in revision; ensure full journal names or abbreviations follow PLOS ONE style).

**Response:** We have reviewed the reference list, ensuring consistency in formatting. All references, including those newly added in this revision, now follow PLOS ONE style, with full journal names or abbreviations correctly applied.

**6.** Confirm that tables and figures have consistent decimal places, units, and legends across the manuscript and supplementary materials.

**Response:** We have reviewed all tables and figures in the manuscript and supplementary materials and made the necessary corrections to ensure consistent decimal places, units, and legends throughout.

**7.** Check the Acknowledgments section for grammatical refinement (e.g., “thank for the commitment” → “thank the researchers for their commitment”).

**Response:** We have revised the Acknowledgments section for grammatical refinement, as below:

*“The authors thank the researchers for their commitment, and the families for their willingness to participate in the study.”*
